# Inorganic polyphosphate occurs in the cell wall of *Chlamydomonas reinhardtii *and accumulates during cytokinesis

**DOI:** 10.1186/1471-2229-7-51

**Published:** 2007-09-24

**Authors:** Thomas P Werner, Nikolaus Amrhein, Florian M Freimoser

**Affiliations:** 1 Institute of Plant Sciences, ETH Zurich, Universitätstrasse 2, CH-8092 Zurich, Switzerland

## Abstract

**Background:**

Inorganic polyphosphate (poly P), linear chains of phosphate residues linked by energy rich phosphoanhydride bonds, is found in every cell and organelle and is abundant in algae. Depending on its localization and concentration, poly P is involved in various biological functions. It serves, for example, as a phosphate store and buffer against alkali, is involved in energy metabolism and regulates the activity of enzymes. Bacteria defective in poly P synthesis are impaired in biofilm development, motility and pathogenicity. PolyP has also been found in fungal cell walls and bacterial envelopes, but has so far not been measured directly or stained specifically in the cell wall of any plant or alga.

**Results:**

Here, we demonstrate the presence of poly P in the cell wall of *Chlamydomonas reinhardtii *by staining with specific poly P binding proteins. The specificity of the poly P signal was verified by various competition experiments, by staining with different poly P binding proteins and by correlation with biochemical quantification. Microscopical investigation at different time-points during growth revealed fluctuations of the poly P signal synchronous with the cell cycle: The poly P staining peaked during late cytokinesis and was independent of the high intracellular poly P content, which fluctuated only slightly during the cell cycle.

**Conclusion:**

The presented staining method provides a specific and sensitive tool for the study of poly P in the extracellular matrices of algae and could be used to describe the dynamic behaviour of cell wall poly P during the cell cycle. We assume that cell wall poly P and intracellular poly P are regulated by distinct mechanisms and it is suggested that cell wall bound poly P might have important protective functions against toxic compounds or pathogens during cytokinesis, when cells are more vulnerable.

## Background

Inorganic polyphosphate (poly P) consists of linear chains of up to several hundred phosphate residues linked by energy rich phosphoanhydride bonds and has been detected in every organism studied so far. The concentration of poly P can vary by many orders of magnitude, even within the same organism. High concentrations of cellular poly P can serve as a phosphate store, as a buffer against alkali (for a review see [1,2]) and are involved in osmoregulation in the algal species *Dunaliella salina *and *Phaeodactylum tricornutum *[3,4]. Low poly P concentrations on the other hand can activate the Lon protease in *E. coli *[5] or the mammalian TOR kinase [6] and affect translation fidelity of ribosomes [5,7]. In humans, poly P modulates blood coagulation and stimulates apoptosis of plasma and myeloma cells [8-10].

PolyP has been found in most cellular compartments such as the nucleus, mitochondria, the cytoplasm and the ER [2,11]. Particularly high concentrations of poly P are stored in fungal vacuoles and in acidocalcisomes of algae and other unicellular organisms [1,12-14]. For example, up to 20% of the *Saccharomyces cerevisiae *dry weight can be accounted for poly P stored in the vacuole [15]. But despite its often very high concentrations, poly P is very difficult to localize specifically. PolyP cannot be fixed, it is water soluble and readily binds to many cellular components during purification. Therefore, it is impossible to exclude contamination of isolated organelles by vacuolar or acidocalcisomal poly P. The specific localization of poly P suffers from additional difficulties: Since poly P lacks structural diversity and occurs ubiquitously, it is not possible to raise antibodies. And stains for polyanionic compounds that are used as poly P dyes, such as toluidine blue (TBO) and 4',6-diamidino-2-phenylindole (DAPI) [16-18], are not specific for poly P: TBO also binds to other polyanionic compounds, as for example nucleic acids, which can lead to similar metachromatic effects as binding to poly P [2,19]. DAPI emits a characteristic yellow fluorescence after binding to poly P that can easily be distinguished from the blue fluorescence of DAPI-DNA complexes [16]. However, the fluorescence intensity of DAPI-poly P complexes is strongly affected by other cellular compounds as for example *S*-adenosylmethionine [16] and binding of DAPI to lipids results in a similar, albeit weaker fluorescence as binding to poly P [20,21]. Recently, we have developed a novel and highly sensitive method for the specific localization of poly P in fungal cell walls [22]. Similar to earlier reports that localized poly P in the vacuoles of *S. cerevisiae *and *Phialocephala fortinii *[24,23], this method employed poly P binding proteins (PBPs) and immunohistochemical detection. Using this method, we were able to establish poly P as a cell wall component of a broad range of fungal species from all phyla [22].

Here we extend these findings from fungi to algae by unequivocally showing the presence of poly P in the cell walls of *Chlamydomonas reinhardtii *and other algae. The cell wall of *C. reinhardtii *consists almost exclusively of 25 to 30 hydroxyproline rich glycoproteins (HPRGs), which are similar to extensins, the major protein component in the cell walls of higher plants [25]. Because *C. reinhardtii *mutants defective in cell wall regeneration are viable, this unicellular algae is used as a model organism to study the proteinaceous fraction of the plant cell wall [26]. The *C. reinhardtii *cell wall is arranged in two major domains. An outer layer consists of a crystalline like matrix of HPRGs, is soluble in chaotropic reagents and probably provides protection against pathogens and mechanical force [27]. The inner layer forms a framework of highly covalently crosslinked HPRGs with a high tensile strength and thus provides resistance to osmotic stress (for a review see [27]). Complex carbohydrates such as cellulose, xyloglucans or β-glucans are completely missing in *C. reinhardtii*, and poly P has not been identified directly and specifically in the extracellular matrix.

In this report we demonstrate by specific staining and biochemical quantification that the cell envelope of *C. rheinhardtii *contains poly P. The content of cell wall localized poly P is very dynamic and reaches the highest levels at the end of cytokinesis. This might imply important functions of cell wall poly P in the algal cell cycle, in cell wall biogenesis or in the resistance against toxins and pathogens during a vulnerable growth phase.

## Results

### Staining of wild type and cell wall mutants with PBPs

**Figure 1:**
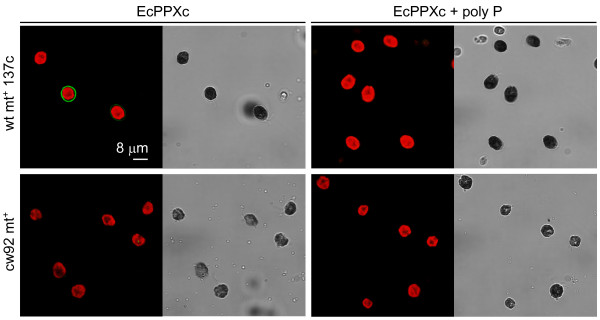
**Cell wall poly P staining of wild type and cell wall deficient *C. reinhardtii *cells**. The confocal microscopic pictures show wild type mt^+ ^137c (above) and cell wall deficient cw92 mt^+ ^(below) *C. reinhardtii *cells that are stained with EcPPXc (green: poly P staining, red: chlorophyll, grayscale: corresponding scattered light picture). Specificity of the poly P staining was controlled by competition with soluble poly P (right). The other wild type strains (mt-137c and mt-CC-410) showed similar staining as wild type mt^+ ^137c (not shown). Because staining of the cell wall mutant strains cw92 mt^+^, cw15 mt^+^, cw15 mt^-^, cw1 mt^-^, cw14 mt^+ ^and cwd mt^- ^led to identical signals, only the cell wall mutant cw92 mt^+ ^is shown as representative.

**Figure 2:**
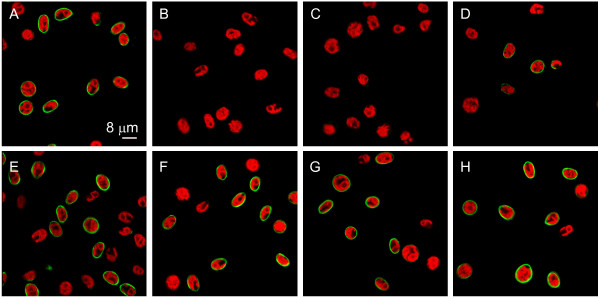
**Competition with various phosphate rich compounds shows specificity of the poly P staining**. The confocal microscopic pictures show poly P staining of wild type mt^+ ^137c cells shortly after cytokinesis with EcPPXc (A) and competition with poly P (B), EcPPXc lacking the MBP tag (C), DNA (D), RNA (E), ATP (F), pyrophosphate (G) and orthophosphate (H) (green: poly P staining, red: chlorophyll).

For detection of poly P in the cell wall of *C. reinhardtii *we used the enzymatically inactive C-terminal domain of the *Escherichia coli *exopolyphosphatase (EcPPXc) as the specific binding protein. EcPPXc was expressed as a fusion protein with a maltose binding protein (MBP) that was used for affinity purification and visualization by immunofluorescence. This method has been used previously to specifically visualize poly P in the cell wall of various filamentous fungi [22]. Asynchronously growing *C. reinhardtii *wildtype cells (mt^+ ^137c, mt^- ^137c, mt^- ^CC-410) were stained with EcPPXc and competition with soluble poly P served as a control for the specificity of the signal. This staining resulted in a clear and strong signal at the cell periphery that was completely inhibited by competition with poly P (Fig. 1). To analyze the specificity of the staining for poly P we added various competitors. No staining of wild type mt^+ ^137c cells was observed upon competition with soluble poly P or another PBP (EcPPXc fused to a GST tag instead of a MBP tag), whereas staining was only weakly reduced by addition of an excess of DNA and not affected at all by the addition of RNA, ATP, pyrophosphate and orthophosphate (Fig. 2). Besides EcPPXc, we also used the ATPase domain of the *E. coli *Lon protease (EcLonA) to detect poly P specifically [22]. Treatment with EcLonA led to similar and specific staining, but the signal was much weaker (not shown). Therefore, EcPPXc was used for all further experiments. To test if the observed signal originated from the cell wall, we used the same protocol to stain cell wall mutant cells (cw15 mt^+^, cw15 mt^-^, cw92 mt^+^, cw1 mt^-^, cw14 mt^+ ^and cwd mt^-^) in the background of the wild type strains used for the initial staining. No fluorescence could be detected at the periphery of any of these mutant strains (Fig. 1).

### Correlation of staining intensity and biochemical quantification of cell wall poly P in *C. reinhardtii *under supply of different phosphate concentrations

**Figure 3:**
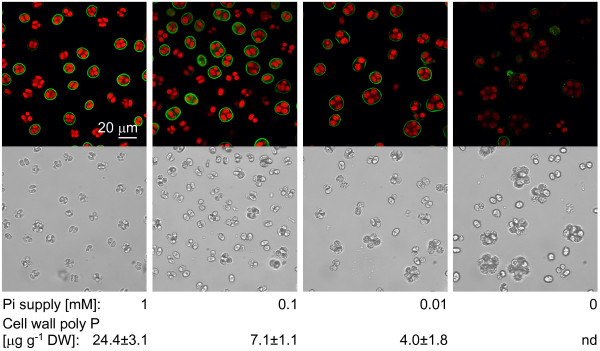
**Correlation of signal intensity and biochemical quantification of cell wall poly P**. Wild type (CC-410) cells were grown in TAP medium supplemented with 1, 0.1, 0.01, and 0 mM Pi, stained for cell wall poly P and analysed by confocal microscopy (green: poly P staining, red: chlorophyll, grayscale: corresponding scattered light picture). Cell wall poly P contents indicated below were quantified biochemically by phosphate release form living cells with a specific exopolyphosphatase (ScPpx1).

Next, we investigated the staining intensity as a function of the phosphate supply in the medium. For this, *C. reinhardtii *wild type strain mt^- ^(CC-410) was grown for 6 days in liquid TAP medium supplemented with 1, 0.1, 0.01 and 0 mM potassium phosphate (pH 7.2). Staining with EcPPXc produced a fluorescent signal from the cell walls the strength of which correlated positively with the phosphate concentration in the medium (Fig. 3). However, fluorescence intensity of chlorophyll was also weaker in cells grown under low phosphate conditions. This might be a consequence of phosphate limitation, but could also be caused by potassium limitation, since potassium phosphate is the only significant source of this cation in TAP medium.

In order to quantify poly P in the cell wall of *C. reinhardtii*, a specific, recombinant exopolyphosphatase from *S. cerevisiae *(ScPpx1) that does not degrade substrates such as ATP or pyrophosphate [28,29], was used to digest poly P directly from the extracellular matrix of living cells. Contamination with poly P or phosphate originating from dead cells was controlled carefully, since *C. reinhardtii *contains high intracellular poly P stores. For this purpose cells were incubated in parallel with and without ScPpx1 and after removing of the cells both extracts were again treated with ScPpx1. Intracellular poly P that was released from dead cells was degraded to orthophosphate in both reactions and the difference in the orthophosphate content should correspond to the cell wall poly P alone. The proportion of orthophosphate released from cell wall poly P was between 12 and 25%. The residual (background) Pi, 75 to 88% of the total Pi measured, originated from intracellular poly P and orthophosphate that were released from cells during the incubation with buffer alone. This method gave a reliable measure for cell wall localized poly P, but the actual content might be underestimated, since a part of cell wall bound poly P chains might be inaccessible for degradation by ScPpx1. The cell wall poly P content reached 24 μg per g dry weight in medium containing 1 mM phosphate (Fig. 3). In cells grown in media containing 0.1 or 0.01 mM phosphate the cell wall poly P content decreased to 7 and 4 μg per g dry weight, respectively (Fig. 3). It was not possible to quantify poly P in cells that were grown in phosphate free medium, although the staining with EcPPXc still produced a faint signal (Fig. 3). These results demonstrated positive correlations between the signal intensity of the staining, the measured poly P content and the supply of potassium phosphate in the medium (Fig. 3).

### Analysis of cell wall bound poly P during the cell cycle

**Figure 4:**
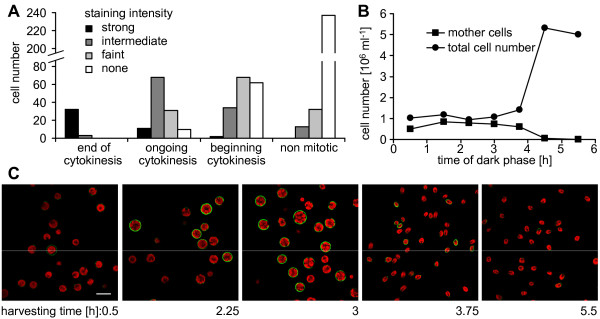
**Correlation of growth phase and poly P staining of the cell wall**. A, Asynchronously growing wild type mt^+ ^137c cells were stained for cell wall poly P. The cells were assigned to four different states of development during cytokinesis and categorized visually according to fluorescence intensity of the cell wall ("strong": Cy2 signal brighter than cholorophyll signal; "intermediate": Cy2 similar or slightly fainter than chlorophyll; "faint": Cy2 visible, but much fainter than chlorophyll; "none": no Cy2 signal visible). B, The numbers of total cells and mother cells at various time points indicate synchronous cell division of wild type mt^+ ^137c cells. C, Cells from this synchronously growing culture were stained at various time points during mitosis and analysed by confocal microscopy (green: poly P staining, red: chlorophyll).

**Figure 5:**
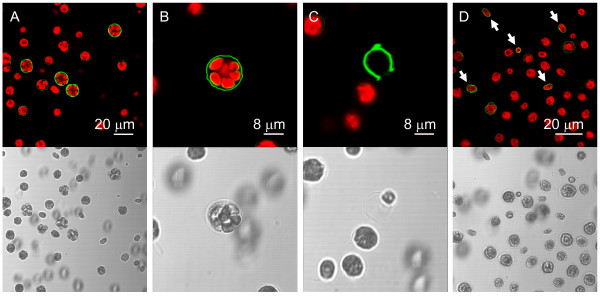
**Poly P staining of mother and daughter cell walls**. Wild type mt^+ ^137c cells were stained with EcPPXc and analysed by confocal microscopy (green: poly P staining, red: chlorophyll). A, Cells during late cytokinesis exhibit the strongest poly P signal. B, Staining of mother and daughter cell walls. C, Strongly stained empty mother cell envelope after release of daughter cells. D, Cell wall staining of freshly released daughter cells (indicated by arrows).

In the wild type strains that were stained with PBPs it appeared that cell walls of mitotic cells emitted a stronger signal. This phenomenon was especially apparent in the strain CC-410, which showed delayed cell separation after mitosis and usually contained more mother cells at the end of cytokinesis. Therefore, we tested whether the poly P content in the cell wall fluctuates during the cell cycle. In a culture of asynchronously growing wild type mt^+ ^137c cells, 91% of the cells that were at the end of cytokinesis stained strongly, whereas at least 61% of the cells in earlier stages revealed an intermediate or faint signal (Fig. 4A). No cell wall signal could be detected in 84% of the non mitotic cells (Fig. 4A, as an example of a stained asynchronous culture see Fig. 5A).

To study the dynamics of the poly P staining during the cell cycle, *C. reinhardtii *wild type mt^+ ^137c cells were grown in synchronous culture and stained at various time points during mitosis. Cells were monitored for synchronous division by counting mother cells (cells with one, two or three visible constrictions) and total number of cells under the light microscope. The sudden increase in total cell number and disappearance of mother cells after about 4 hours in the dark phase indicated simultaneous release of daughter cells and thereby synchronous cytokinesis (Fig. 4B). PolyP staining of cells from this synchronous culture at different time-points showed the strongest signal after 3 h in the dark (Fig. 4C). At this time point, most cells had reached the final state of cytokinesis, just before the release of the daughter cells (Fig. 4B). At the 2.25 h time-point, only few cells showed an intense cell wall signal, and at the 0.5 h point almost no fluorescence was detected (Fig. 4C). Interestingly, the cell walls of about one third of the daughter cells were stained clearly shortly after release from the mother cell, but the stain faded almost completely during the following 1.75 h (Fig. 4C). Microscopical analysis of individual cells with higher magnification revealed indeed both, staining of the mother cell envelope before (Fig. 5B) and after release of the daughter cells (Fig. 5C) and staining of the cell walls of daughter cells within the envelope of the mother cell (Fig. 5B) and after their release (Fig. 5D).

**Figure 6:**
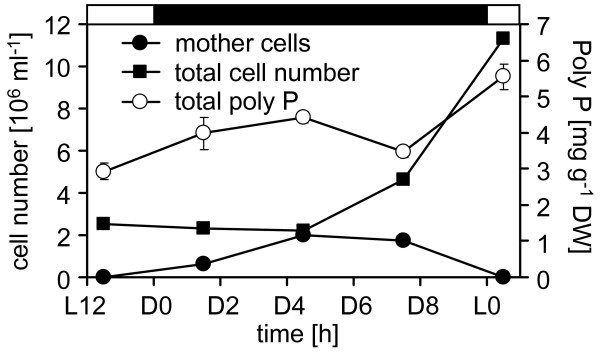
**Total poly P content during mitosis of *C. reinhardtii***. Synchronous growth of a wild type mt^+ ^137c culture was monitored by counting total cell and mother cell number, respectively, at various time points during mitosis, and at the same time cells were harvested for biochemical poly P quantification.

The same synchronously growing wild type mt^+ ^137c cells that were stained were also used to quantify total cellular poly P levels during cytokinesis. Interestingly, the total poly P content did not change drastically during the cell cycle (Fig. 6), but revealed only a slight peak at the end of cytokinesis and doubled slowly during the dark phase from about 2.9 mg/g DW to 5.5 mg/g DW (Fig. 6).

## Discussion

We have detected poly P in the cell wall of *C. reinhardtii *by staining with proteins that specifically bind poly P and by biochemical quantification with a specific recombinant polyphosphatase. This is, to our knowledge, the first report that identifies poly P in the cell wall of any plant species by direct labelling with a specific binding protein or by biochemical quantification. To unequivocally demonstrate the presence of poly P in the extracellular matrix of *C. reinhardtii*, the same criteria for a specific poly P staining were fulfilled as before for fungal cell walls [22]: (1) The staining is reduced by addition of poly P or other poly P binding proteins, but not by an excess of other phosphate containing components (DNA, RNA, ATP, pyrophosphate or phosphate), (2) the staining intensity correlates with the biochemical poly P quantification and (3) application of different PBPs results in staining of the same structures. The immunohistochemical staining of *C. reinhardtii *with specific PBPs fulfilled all of these criteria and is therefore considered to provide proof for the presence of poly P in the cell wall of this alga. This conclusion was further confirmed by the complete absence of any poly P signal in *C. reinhardtii *mutants lacking a cell wall.

Surprisingly, cell wall bound poly P showed a very dynamic behaviour, and accumulated drastically for a short time period during late cytokinesis. At the time point of strongest staining, the daughter cells appeared to be completely separated from each other and to be ready for release from the mother cell envelope. Interestingly, not only the mother cell envelope but also newly synthesized daughter cells were stained for a short time period after their release. This finding led us to conclude that the few and small, non mitotic cells in asynchronous cultures that emitted a clear cell wall signal, were in fact freshly released daughter cells.

There is no relationship between the drastic changes of cell wall and total cellular poly P, respectively, as total poly P levels increased only slightly during cytokinesis and increased again slowly towards the end of the dark phase. Therefore, we assume that the low levels of cell wall poly P and the high levels of intracellular poly P stored in acidocalcisomes are regulated by distinct mechanisms. The location of poly P synthesis remains unclear. Since the poly P rich vacuolar inclusions topologically correspond to the extracellular matrix, it could be assumed that poly P reaches the cell wall by secretory vesicles. However, direct synthesis of poly P in the cell wall upon secretion of enzymes and substrates could be considered as well.

**Figure 7:**
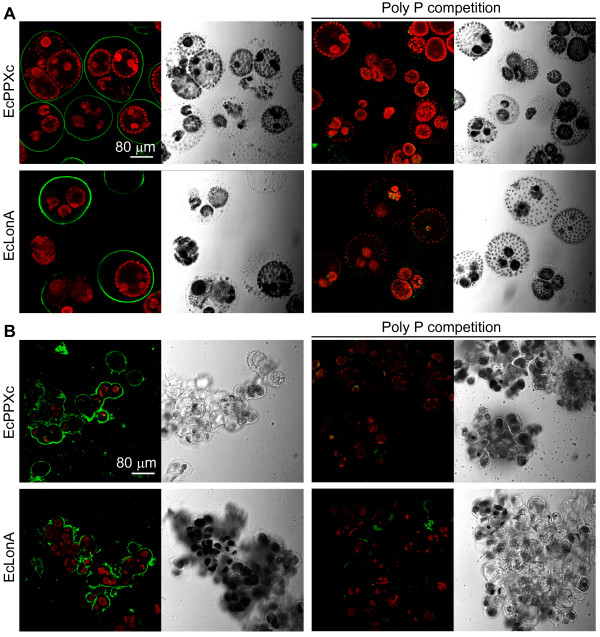
**Staining of cell wall poly P of *Volvox aureus *and *Coleochaete scutata***. The confocal microscopic pictures and corresponding scattered light pictures show *Volvox aureus *and *Coleochaete scutata *cells (green: poly P staining, red: chlorophyll). Both algae were stained with EcPPXc and EcLonA (left) and the specificity of the poly P staining was controlled by competition with soluble poly P (right).

Cell wall bound poly P is not peculiar to *C. reinhardtii*, as we found specific poly P staining with PBPs in two other green algae, i.e. *Volvox aureus *and *Coleochaete scutata *(Fig. 7), and two earlier studies also suggested the existence of cell wall bound poly P in algae. In *Chlorella fusca*, the occurrence of extracellular poly P was deduced from a shift in the poly P peak of ^31^P-nuclear magnetic resonance (^31^P NMR) spectra upon high pH or high ethylene-diamine-tetraacetic acid (EDTA) concentration in the external medium [30]. And *Chlamydomonas acidophila *revealed a signal at the cell periphery after treatment with the unspecific staining agent DAPI, when phosphate-starved cells were transferred to high phosphate media [31]. PolyP has been identified as a cell wall component in a broad range of bacterial and fungal species [22,32,33]. The diverse environments of these organisms and the high variation in poly P concentrations imply different biological roles of this polymer.

High poly P contents have been found, for example, in the cell wall of Mucoralean species, from where poly P is remobilized under low Pi conditions to serve as phosphate supply [22]. *C. reinhardtii *also was shown to secrete phosphatases under low phosphate conditions [34]. However, considering the high poly P content of acidocalcisomes, the poly P content of the cell wall of *C. reinhardtii *appears to be too low to serve a similar function.

Cell wall bound poly P might also protect against the toxic effects of heavy metals [2]. It has been proposed that binding of toxic metals to the cell wall reduces their entry into the cell in algal and fungal species [35,36] and wall less mutants of *C. reinhardtii *indeed have a higher sensitivity towards heavy metals [36]. Due to the ability of poly P to form complexes with various metal ions [1], it is reasonable to assume that cell wall poly P might at least partially be responsible for the retention of heavy metals.

On the other hand, it has been proposed that poly P might act as scavenger for nutrient ions in the cell envelope of the bacterial pathogen *Neisseria meningitides *[33]. Consequently, it would be interesting to investigate if phosphate starved *C. reinhardtii *cells are more susceptible to metal deficiencies. At the same time, the chelation of essential cations in the cell wall could also be a strategy to limit growth of pathogens and other algal species and thereby reduce competition. This hypothesis is supported by the potent antimicrobial activity of poly P against bacteria and fungi that is based on the complexation of divalent cations in the medium by poly P [37,38].

Assuming protective properties of poly P, its presence might be especially important during the delicate state of cytokinesis, when the protective cell wall of the mother cell is degraded and the cell wall of the daughter cells has not yet fully formed. This shielding against toxic compounds and pathogens during a vulnerable phase of the cell cycle might be a biological explanation for high poly P levels in the cell wall during cytokinesis.

## Conclusion

In this report we established a staining method that provides a sensitive and specific tool for the study of poly P in the extracellular matrices of algal species. We used this method to demonstrate the presence of poly P in the cell wall of *C. reinhardtii *and two other algal species. Signal intensity of cell wall bound poly Pshowed a very dynamic behaviour and was highest at the end of cytokinesis. Because this was in contrast to the rather constant intracellular poly P stores, we assumed different regulatory mechanism for both poly P pools. This selective appearance of poly P during late cytokinesis might imply an important role of poly P in cell wall biogenesis or protective functions of poly P during this vulnerable phase of the cell cycle.

## Methods

### Strains and culture conditions

The following *Chlamydomonas reinhardtii *strains were all obtained from the Chlamydomonas Genetics Center (Duke University, Durham, NC USA): CC-410 wild type mt^-^, CC-124 wild type mt^- ^137c, CC-125 wild type mt^+ ^137c, CC-400 cw15 mt^+^, CC-3491 cw15 mt^-^, CC-503 cw92 mt^+^, CC-846 cw1 mt^-^, CC-847 cw14 mt^+ ^and CC-2656 cwd mt^-^. *Volvox aureus *(88-1) and *Coleochaete scutata *(110.80 M) were obtained from the Culture Collection of Algae (SAG) of the University of Göttingen (Göttingen, Germany). All algal species were kept on 2% TAP agar plates [39]. *Chlamydomonas reinhardtii *cells were grown to the end of the exponential phase (cell density between 10^6 ^and 2 × 10^7 ^cells per ml) in 250 ml Erlenmeyer flasks containing 50 ml TAP medium on a rotating platform (90 rpm) under 16/8-h light/dark cycles (1700 μmol m^-2 ^s^-1^, 24°C).

Wild type mt^+ ^137c (CC-125) cells were synchronized in HSM medium [40,41] supplemented with 0.12% sodium acetate trihydrate and 0.4% yeast extract under continuous magnetic stirring and 14/10-h light/dark cycles (750 μmol m^-2 ^s^-1^, 24°C). Three ml starting cultures were inoculated with cells from TAP agar plates and grown to stationary phase for 6 days. One hundred μl of these starting cultures were used to inoculate 50 ml precultures in 250 ml Erlenmeyer flasks at the beginning of a light period. These precultures were kept for at least four light/dark cycles or until a cell density of 10^7 ^cells per ml had been reached. Cultures of 50 ml were inoculated with 7 ml synchronized cells at the beginning of a light period and used for analysis during the next dark period, when they passed through cytokinesis.

Cell numbers were determined by counting in a Neubauer chamber (Neubauer improved, Omnilab AG, Mettmenstetten, Switzerland) after addition of paraformaldehyde to a final concentration of 1%. At least 150 cells were counted for the calculation of cell densities. Four different cell types that occur during cytokinesis were distinguished: (1) Beginnning of cytokinesis (large, round cells showing an amorphous structure and eventually signs of a starting division), (2) ongoing cytokinesis (one, two or three clearly visible constrictions), (3) end of cytokinesis (mostly eight clearly separated daughter cells surrounded by the mother cell envelope), (4) non mitotic cells (small cells with an oval shape showing no signs of division).

### Staining of poly P in cell walls for fluorescence microscopy

Poly P was stained using the C-terminus of the exopolyphosphatase (EcPPXc) fused to a maltose binding protein (MBP) tag and a His tag. The corresponding gene was cloned and the recombinant protein purified using the MBP tag for affinity chromatography as described before [22]. Algal cells were always pelleted by centrifugation at 2'300 *g *for 1 min in 1.5 ml tubes and incubation was performed at room temperature under slow overhead rotation to prevent sedimentation. Staining was carried out as described before with some modifications [22]. *Chlamydomonas reinhardtii *cell-suspensions of 0.2 *OD*_680 _units (wild type) or 0.5 *OD*_680 _units (cell wall mutants) were pelleted, resuspended in 80 μl blocking buffer (1% BSA in low salt PBS: 0.4 mM KH_2_PO_4_, 1.6 mM NaH_2_PO_4_, 10 mM NaCl, pH 7.3) and incubated for 15 min. *Volvox aureus *and *Coleochaete scutata *were scratched from TAP agar plates and blocked in the same way. The cells were washed twice (washing was always done with 80 μl low salt PBS) and incubated with 80 μl PBPs (at least 20 min, 0.7 μM PBP in blocking solution). For competition experiments, 17 μM poly P (Sigma-Aldrich Chemie Gmbh, Steinheim, Germany; the concentration was calculated assuming an average chain lenght of 88 phosphate residues), 7 μM of EcPPXc fused to a GST tag and a His tag (for cloning and purification see [22]) or 1.5 mM DNA, RNA, ATP, pyrophosphate or inorganic phosphate (concentration based on phosphate residues) were added. The samples were washed three times, fixed (20 min, 80 μl, 4% paraformaldehyde in low salt PBS), washed and blocked again (20 min). After washing, cells were incubated with 80 μl primary antibody (1 μg/ml monoclonal anti MBP antibody (New England Biolabs, Beverly, MA USA) in blocking solution). After washing (three times) 80 μl of secondary antibody was added (1 h, 7.5 μg/ml Cy2 labeled goat anti-mouse IgG, Jackson Immuno Research, West Grove, PA USA). After three final washes, the cells were diluted in low salt PBS and 2.5 μl were mounted on Teflon-coated 10 well slides (Menzel GmbH & Co KG Braunschweig, Germany). The microscopical pictures were taken with a confocal laser scanning microscope (Leica DM IRBE and Leica TCS SP laser; Leica, Unterentfelden, Switzerland) using an ArKr laser at λ = 476 nm for excitation. Fluorescence of Cy2 and chlorophyll was detected from λ = 490 to 540 nm and from λ = 660 to 750 nm, respectively. Pictures of the stained samples and their controls were taken with identical settings and pictures were not processed digitally except for overlay of different channels and contrast adjustments by identical numerical values.

### Quantification of cell wall bound poly P

*Chlamydomonas reinhardtii *wild type cells (CC-410) were grown in TAP medium containing 0.01 mM, 0.1 mM and 1 mM phosphate to a density of about 5 × 10^6 ^(0.01 mM Pi) and 10^7 ^(0.1 and 1 mM Pi) cells per ml. Cells from a total culture volume of 100 ml (0.01 mM and 0.1 mM Pi) or 50 ml (1 mM Pi) were harvested (cells were always centrifuged for 2 min at 2'300 *g*) and washed twice with 25 ml PPX buffer (50 mM Tris, 5 mM MgCl_2_, pH 7.6). They were resuspended in 10 ml PPX buffer, split into nine equal aliquots and pelleted. Three samples were frozen in liquid nitrogen and lyophilized (20 Pa, -20°C, 24 h) for dry weight determination. Three pellets were suspended in 80 μl PPX buffer containing 2.5 × 10^6 ^U (one unit corresponds to the release of 1 pmol Pi per min at 37°C) of a recombinant exopolyphosphatase from *Saccharomyces cerevisiae *(ScPpx1) [29,42]. The final three pellets were resuspended in PPX buffer without enzyme. After incubation (37°C, 20 min, gentle shaking every 5 min) cells were again pelleted and 50 μl of the supernatant was collected. For discrimination between phosphate released from cell wall bound poly P and phosphate originating from intracellular poly P, 80 μl reaction buffer containing 2.5 × 10^6 ^U ScPpx1 were added to all six samples followed by incubation for 20 min at 37°C. The released phosphate was quantified as described (Werner et al. 2005).

### Purification and quantification of total poly P

For determination of total cellular poly P, 1*OD*_680 _unit of cells was harvested (2'300*g*, 2 min) and the pellet frozen immediately at -20°C for later analysis. Upon thawing, cells were extracted with 50 μl of 1 M H_2_SO_4_, poly P was purified on PCR purification columns and enzymaticaly digested with ScPpx1, and the released phosphate was colorimetrically quantified exactly as described before [42]. However, the total poly P contents might be underestimated due to reduced binding of low poly P concentrations and short poly P chains to the silica membranes [42].

### Reproducibility and statistics

All experiments were repeated at least twice. Every data point shown in the figures represents an average value obtained from three individually analyzed samples. Error bars and deviations are indicated as standard errors. Error bars that are not visible were smaller than the symbols representing the average values.

## Competing interests

The author(s) declares that there are no competing interests.

## Authors' contributions

TPW carried out the experimental work and the data analysis, participated in the design of the study and drafted the manuscript. NA participated in the design of the study and revised the manuscript critically for important intellectual content. FMF carried out the design of the study and drafted the manuscript. All authors read and approved the final manuscript.
